# Striving for Consensus: Neuroma Prevention and Preferred Approaches in Surgical Digit Amputations—A Survey of Hand Surgeons

**DOI:** 10.3390/jcm15135300

**Published:** 2026-07-07

**Authors:** Alexander Draschl, Werner Girsch, Lars-Peter Kamolz, Patrick Sadoghi, Juergen Dolderer, Marcel Hoh, Sebastian P. Nischwitz

**Affiliations:** 1 Department of Plastic and Hand Surgery, University Medical Center Bayreuth, Friedrich-Alexander University Erlangen-Nuremberg, 95445 Bayreuth, Germany; 2 Research Unit for Responsible Aesthetics, Division of Plastic, Aesthetic and Reconstructive Surgery, Department of Surgery, Medical University of Graz, 8010 Graz, Austria; 3 Division of Plastic, Aesthetic and Reconstructive Surgery, Department of Surgery, Medical University of Graz, 8010 Graz, Austria; 4 Department of Orthopedics and Trauma, Medical University of Graz, 8010 Graz, Austria

**Keywords:** stump formation, neuroma, flexor tendon, extensor tendon, digital amputation, amputation injuries, hand surgery, traumatic finger injuries

## Abstract

**Background**: Although surgical digit amputations are common procedures in hand surgery, substantial differences in technical approaches have been reported, with the development of symptomatic neuromas posing a major challenge. Given the absence of a universally accepted technique, especially concerning painful neuroma prevention, this survey study’s rationale was to explore the preferred surgical approach for traumatic noninfected digital amputation injuries (excluding the thumb) at the interphalangeal (IP) joint levels. **Methods**: A 10-point online questionnaire was sent to ÖGH, DGH and SGH members. To provide a comprehensive overview of the preferred approach, descriptive statistics were performed, while examining differences based on years of surgical experience (≤20 years versus >20) and society affiliation (ÖGH vs. DGH vs. SGH) via inferential Pearson-chi square and Fisher’s exact tests were employed. **Results:** Of the 1670 experts contacted, 213 (12.8%) took part in the survey. Across different societies and years of surgical experience, there is a consensus on most aspects, including smoothing bony edges after transosseous resection (99.8%), removal of articular cartilage after disarticulation (78.9%), shortening flexor tendons (81.2%), and avoiding additional extensor tendon shortening (92.3%). No consensus was found on the technique of surgical bone transection, the reduction of the phalangeal head during disarticulation and the treatment of digital nerves including neuroma prevention. **Conclusions**: Although there is a broad consensus on most aspects of surgical digit amputation, the treatment of digital nerves and neuroma prevention remains an area with a lack of consensus. Future studies should focus on these aspects to uncover further benefits.

## 1. Introduction

Digital amputation injuries are one of the most common injuries that can have profound and far-reaching consequences for individuals of all age groups [1,2]. These consequences include psychological and physical sequelae, encompassing social withdrawal, reduced quality of life, limited functionality, and stigma experienced [3,4,5]. In addition, these injuries can impose an economic burden, especially on working men, manifesting as income loss and obstacles to future earnings [1,2,5,6,7].

As one of the consequences with profound impact, the development of painful neuromas following amputation injuries remains a major concern for both patients and surgeons [8,9,10,11]. On the one hand, studies indicate that 2.7% to 30% of patients experience painful neuromas after digital amputation injuries [11,12,13,14]. On the other hand, the absence of a universally accepted approach is evident in the wide range of surgical and non-surgical techniques (>150 methods) for managing and preventing symptomatic neuromas [9,15].

While the initial surgical management of the transected nerve is considered the cornerstone for minimizing neuroma formation, non-surgical treatment modalities also play an important role within a multidisciplinary treatment strategy [15]. Current conservative options include pharmacological therapies such as gabapentinoids (gabapentin, pregabalin), tricyclic antidepressants, and serotonin-norepinephrine reuptake inhibitors (SNRIs), as well as topical agents including lidocaine and capsaicin [15]. Furthermore, hand therapy interventions such as scar massage, desensitization techniques, therapeutic ultrasound, and transcutaneous electrical nerve stimulation (TENS) have been reported to alleviate neuropathic symptoms and improve patient function [15,16]. Therefore, both operative and non-operative approaches should be considered when developing an individualized treatment plan for patients with painful digital neuromas [15,17].

In general, there are two surgical methods for treating amputated or devascularized digits: (a) replantation and (b) surgical amputation [8,18,19,20]. While replantation may be a viable option in some instances, it is not always feasible or able to provide satisfactory functional outcomes [21,22,23,24,25,26]. Surgical digit amputation, however, is commonly performed for traumatic digital injuries, especially when multiple digits are affected [21,23], and it can offer socioeconomic benefits, including a faster return to work and simplified intraoperative and postoperative management compared to replantation [18,23,27].

A survey study of American hand surgeons revealed substantial variations in surgical approaches for noninfected traumatic digital amputations at the interphalangeal (IP) joint levels [8]. Interestingly, the potential of centro-central union (CCU) in preventing painful neuromas, an approach with promising results [28,29,30], has been overlooked by hand surgeons in their study, with the majority favoring to place the nerve endings away from the incision site or anticipated area of highest pressure [8]. Notably, no comparable study has been conducted in German-speaking countries.

Therefore, this survey study’s objective was to investigate current preferences and practice patterns among hand surgeons regarding the surgical approaches employed for digit amputation in noninfected traumatic digital amputation injuries at the IP levels.

## 2. Materials and Methods

The study adhered to the principles of the Declaration of Helsinki and the International Conference on Harmonization-Good Clinical Practice (ICH-GCP). The study protocol was approved by the responsible institutional ethics committee (EK number: 34-050 ex 21/22).

### 2.1. Study Design and Population

An online questionnaire was created in German and uploaded to the electronic survey platform SurveyMonkey (SurveyMonkey Europe UC, Dublin, Ireland). A link to the voluntary and anonymous survey was sent to members of the Austrian Society for Surgery of the Hand (Österreichische Gesellschaft für Handchirurgie, ÖGH), German Society for Hand Surgery, (Deutsche Gesellschaft für Handchirurgie, DGH), and Swiss Society for Hand Surgery (Schweizerische Gesellschaft für Handchirurgie, SGH). The sole inclusion criterion was membership in at least one addressed society. These recipients were chosen, ensuring a competent and knowledgeable group was targeted for participation. The online questionnaires were sent on 10 January 2022 and were utilized for statistical analysis until the end of February 2022 (28 February 2022).

### 2.2. Questionnaire

The 10-point online questionnaire used in the survey study addressed the surgical approach to digital amputation injuries without the option of replantation in cases of traumatic, noninfected digital amputation injuries, specifically at the level of the IP joints. The questionnaire included inquiries regarding functional considerations, management of bony structures, tendons, and digital nerves, with a particular emphasis on techniques for preventing symptomatic neuromas (Table 1). Demographic variables were collected to characterize the study population; “surgical experience” and “affiliated society” were additionally analyzed as potential factors influencing treatment preferences.

### 2.3. Outcomes

The primary outcome of the study was to determine whether a consensus exists in the respective areas concerning the surgical approach. As secondary outcomes, the data were analyzed to examine differences in participant responses based on society affiliation and surgical experience (≤20 years vs. >20 years).

### 2.4. Statistical Analysis

Descriptive statistics were performed using Microsoft^®^ Excel (Version 16.57). For inferential statistics, Pearson-chi square and Fisher’s exact tests with the expected value based on Cramer-V (φc) for interpretation were employed due to the nominal nature of the data, and the statistics software SPSS (Statistical Package of Social Sciences, Version 27) was used. A *p* < 0.05 was considered statistically significant. In cases where a statistically significant difference was detected, a Bonferroni correction was applied, if necessary, to determine the specific column proportions (responses) that exhibited significance. Consensus was defined as results with ≥75% agreement [31].

## 3. Results

Between January 2022 and February 2022, approximately 1670 members (ÖGH: *n* = 257; DGH: *n* ≈ 1200; SGH: *n* = 213) of the respective hand surgery societies in the three main German-speaking countries were addressed. The overall response rate was 12.8%, with 213 experts participating in the survey. The response rate of ÖGH members was 27.6%, and that of SGH members was 14.1%. DGH members had the lowest response rate (≈9.3%).

### 3.1. Demographics

Table 2 displays the demographic characteristics of the study population. There were no significant differences observed in the baseline characteristics of the hand surgeons based on their membership, except for surgical experience: More DGH (*n* = 44, 40.4%) and SGH (*n* = 13, 44.8%) members had 11–20 years of surgical expertise than ÖGH (*n* = 13, 18.6%) members (*p* = 0.014) (Table 3). The distribution of respondents per question and the overall response rate for each question are presented in Table 4.

### 3.2. Aspects Without Consensus

#### 3.2.1. Disarticulation vs. Resection Through Bone (Transosseous Resection) (Q2)

**Figure 1:**
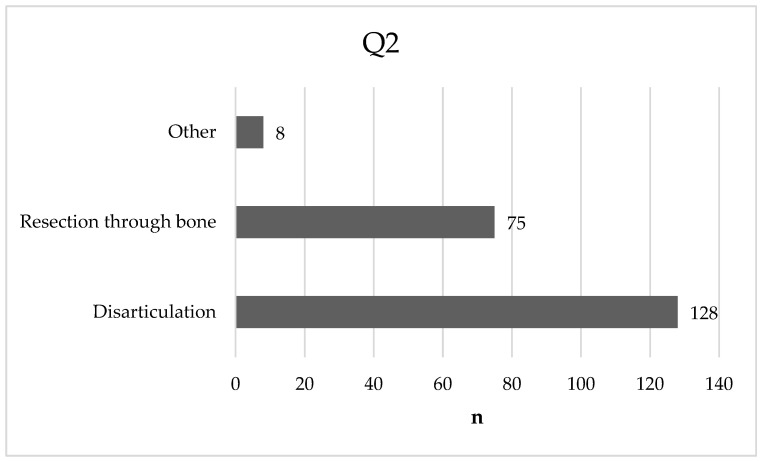
The preferred approach regarding disarticulation versus resection through bone (Q2: “Which of the specified techniques for surgical digit amputation do you perform for non-infected traumatic amputation injuries at the level of the interphalangeal joints (IP)?”).

Most respondents (*n* = 128, 60.7%) expressed a preference for disarticulation, while 75 hand surgeons (35.6%) favored resection through bone as their preferred approach (Figure 1). Eight hand surgeons (3.8%) provided responses that deviated from the available options, hence categorized as *Other*. Among these, four experts (1.9%) indicated that their choice of technique would depend on the soft tissue situation and the level of injury. Another four experts (1.9%) reported that they would opt for disarticulation with either beveling of the trochlear edges (*n* = 1), resection of the condyles (*n* = 1), or removal of the residual articular cartilage combined with resecting the condyles (*n* = 1).

#### 3.2.2. Disarticulation and the Phalangeal Head (Q5)

**Figure 2:**
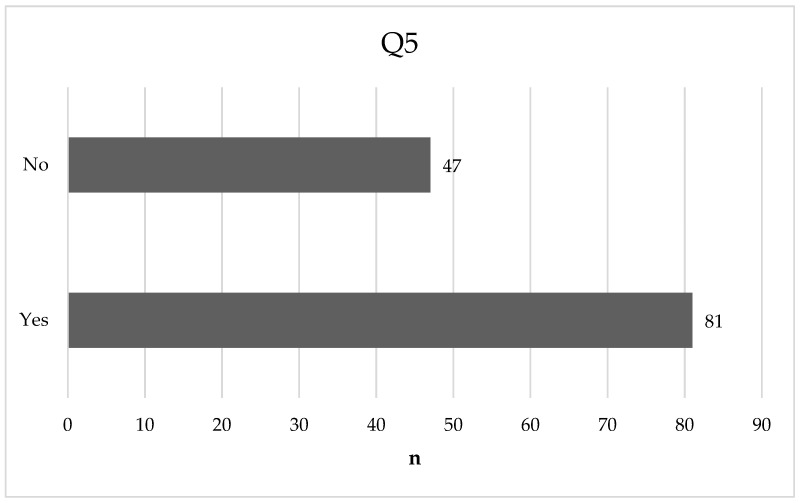
Preference regarding size reduction of the residual phalangeal head following disarticulation (Q5: “Do you reduce the size of the residual phalangeal head during disarticulation?”).

The majority (*n* = 81, 63.3%) reported reducing the size of the residual phalangeal head during IP joint disarticulation. In contrast, 36.7% (*n* = 47) stated that they would not perform any reduction of the phalangeal head (Figure 2).

#### 3.2.3. Management of Digital Nerves (Q6 & Q7)

**Figure 3:**
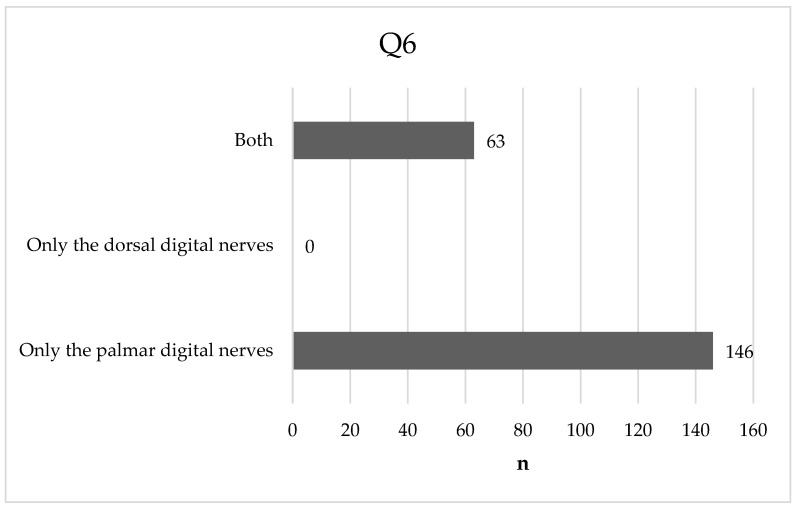
Preference regarding which digital nerves to consider/treat (Q6: “Do you consider/treat only the palmar digital nerves or also the dorsal digital nerves during surgical digit amputation?”).

Nearly a third of the experts (*n* = 63, 30.1%) reported considering and treating both the dorsal and palmar digital nerves. In contrast, most respondents (*n* = 146, 69.9%) stated considering and treating only the palmar digital nerves. Notably, none of the surveyed hand surgeons reported solely considering/treating the dorsal digital nerves (Figure 3).

**Figure 4:**
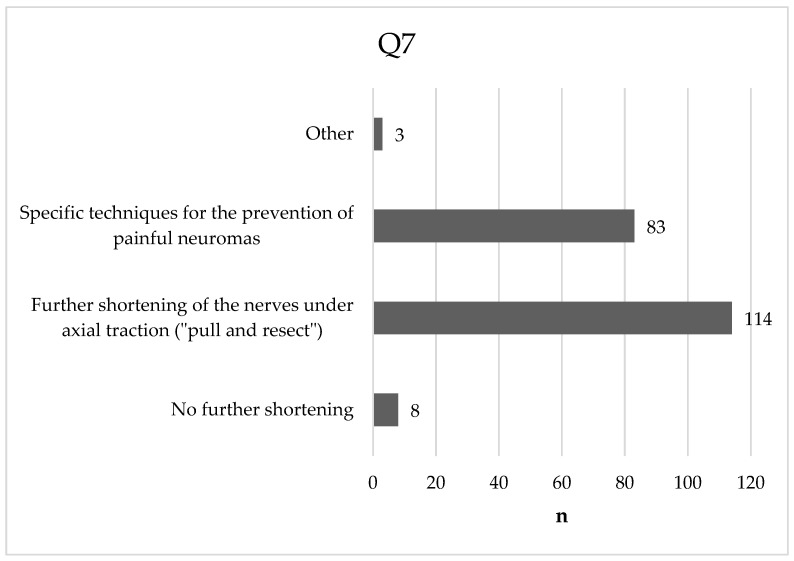
Preference regarding digital nerve management (Q7: “How do you treat the digital nerves of the affected fingers?”).

Regarding Q7, most respondents (*n* = 114, 54.8%) reported further shortening of the nerves under axial traction using the “pull and resect” technique. Additionally, 39.9% (*n* = 83) indicated using specific techniques to prevent painful neuromas. A smaller proportion (*n* = 8, 3.9%) stated that they do not perform any further shortening of the digital nerves (Figure 4). Three participants provided alternative responses, including techniques such as coagulation of the nerve endings (ÖGH member), resecting the nerve (DGH member), and using the “pull and resect” technique with electrocoagulation (SGH member).

#### 3.2.4. Prevention of Painful Neuromas (Q8)

**Figure 5:**
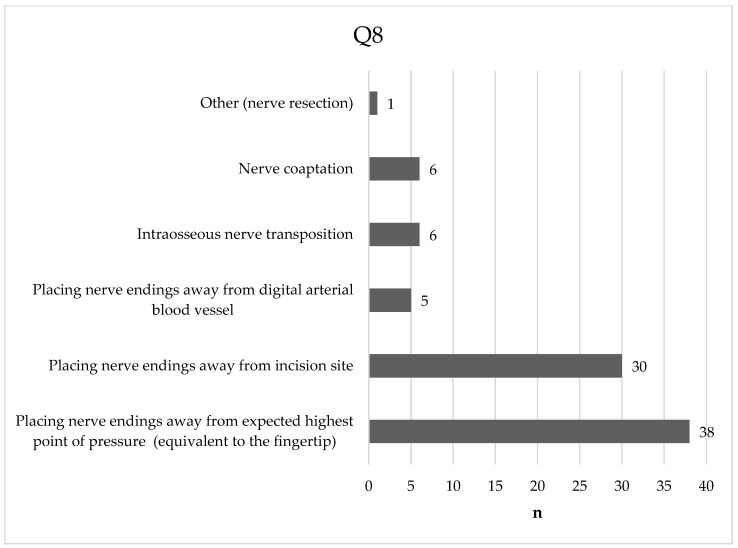
Preference regarding prevention of painful neuroma formation (Q8: “Which of the following techniques do you use to prevent painful neuromas during surgical digit amputation?”).

The majority (*n* = 38, 44.2%) prefers placing the nerve endings away from the anticipated area of highest pressure (equivalent to the fingertip), followed by 30 out of 86 participants (34.9%) preferring to place the nerve endings away from the incision site. Two response options received equal support from 7.0% of experts (six individuals each). The first option involved intraosseous nerve transposition, while the second involved coapting nerve endings (“looping”). Five hand surgeons (5.8%) expressed the approach of separating the nerve endings from the digital arterial blood vessel. Furthermore, one DGH member (1.2%) provided a free text response with nerve resection as their preferred method (Figure 5).

### 3.3. Areas with Consensus

#### 3.3.1. Functional Aspects (Q1)

**Figure 6:**
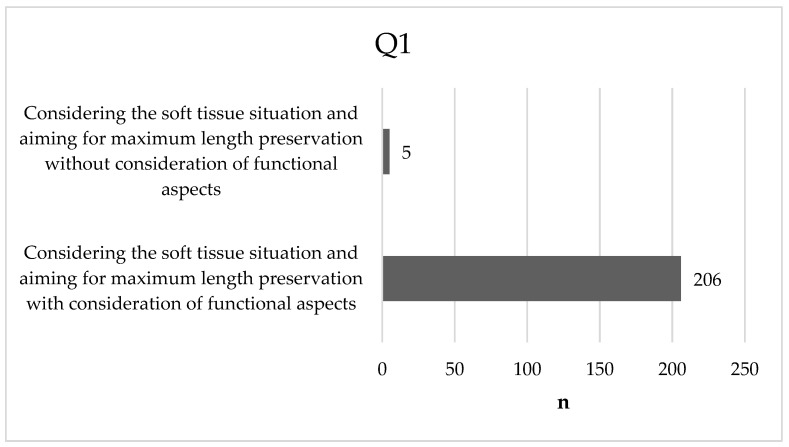
Preference regarding consideration of functional aspects in addition to the soft tissue situation and maximum length preservation (Q1: “Do you perform surgical digit amputation solely considering the soft tissue situation and aiming for maximum length preservation, or do you also consider functional aspects?”).

Among 211 respondents, 97.6% (*n* = 206) indicated performing surgical digit amputation while considering functional aspects in addition to the soft tissue condition and maximizing length preservation. Conversely, two experts (2.4%) reported performing surgical digit amputation solely based on the soft tissue situation and maximum length preservation without considering functional aspects (Figure 6).

#### 3.3.2. Transosseous Resection and Bone Smoothing (Q3)

**Figure 7:**
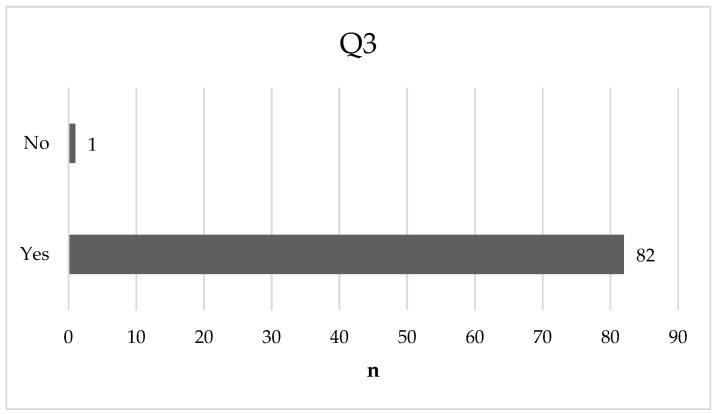
Preference regarding smoothing bony edges following transosseous resection (Q3: “Do you perform an additional smoothing of the bone edges during resection through bone?”).

Among the 83 study participants who additionally answered Q3, 82 (98.8%) included bone edge smoothing as a component of their transosseous resection technique, and one expert (1.2%) indicated not to do so (Figure 7).

#### 3.3.3. Disarticulation and Articular Cartilage (Q7)

**Figure 8:**
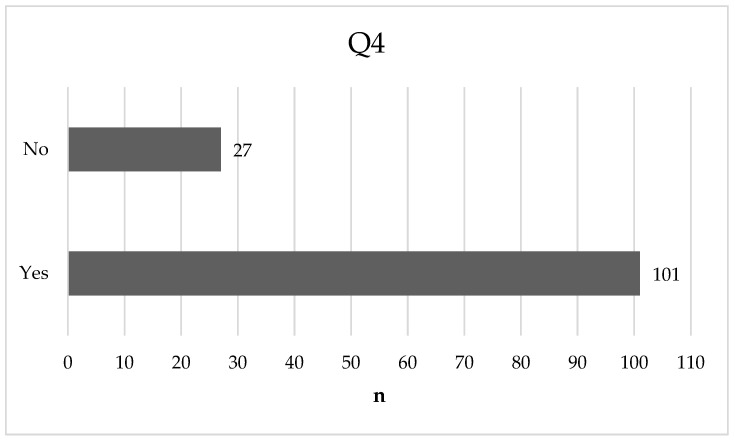
Preference regarding removal of the articular cartilage following disarticulation (Q4: “Do you remove the articular cartilage during disarticulation?”).

Among the 128 individuals who expressed a preference for disarticulation, the majority (*n* = 101, 78.9%) stated that they would remove the residual articular cartilage, while 27 hand surgeons (21.1%) reported not doing so (Figure 8).

#### 3.3.4. Management of Flexor Tendons (Q9)

**Figure 9:**
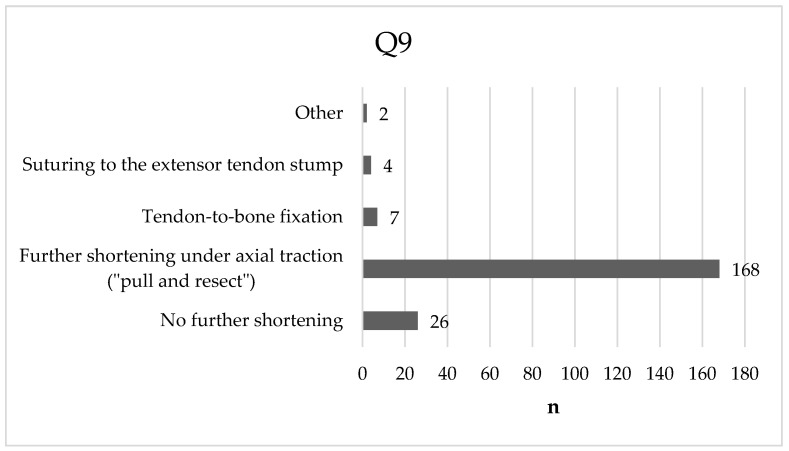
Preference regarding flexor tendon management (Q9: “How do you manage the flexor tendons?”).

The majority (*n* = 168, 81.2%) reported further shortening of the flexor tendons via “pull and resect” technique. While 12.6% (*n* = 26) of the hand surgeons stated not performing further extensor tendon shortening, 3.4% (*n* = 7) favored suturing the flexor tendon to the extensor tendon stump. Additionally, two individuals (1.0%) provided alternative free-text responses: One DGH member described the technique of fixing the FDS tendon to the bone to preserve mobility in the PIP joint while alternatively considering complete resection; an SGH member described a variable approach, where the flexor tendon’s fixation, shortening, or suturing is chosen depending on the specific situation (Figure 9).

#### 3.3.5. Management of Extensor Tendons (Q10)

**Figure 10:**
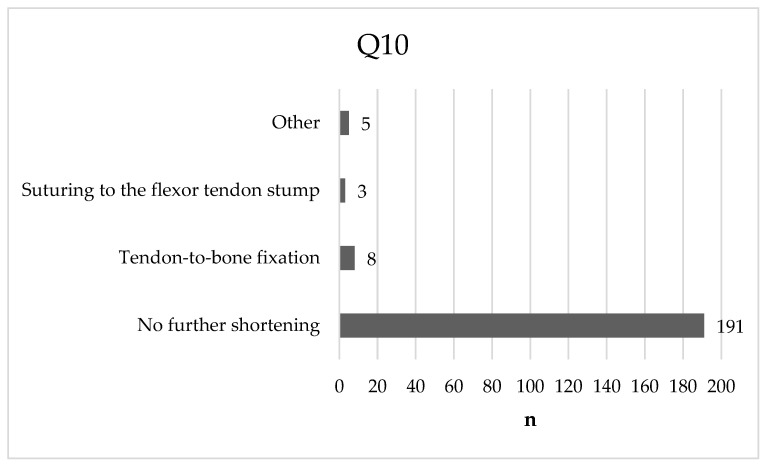
Preference regarding extensor tendon management (Q10: “How do you manage the extensor tendons?”).

Most experts (*n* = 191, 92.3%) indicated not performing any further extensor tendon shortening. In contrast, 3.9% (*n* = 8) of the participants opted for tendon-to-bone fixation, while 1.5% (*n* = 3) reported to favor suturing the extensor tendon to the flexor tendon stump (Figure 10).

### 3.4. Differences Based on Society Affiliation (ÖGH vs. DGH vs. SGH)

Statistically significant differences based on society affiliation were found in the approach of bone transection (Q2), consideration of digital nerves (Q6), and the management of both flexor and extensor tendons (Q9–10) (Table 5).

Regarding Q2, there were no statistically significant differences in responses pertaining to *Disarticulation* and *Resection through bone (transosseous resection)*. However, a significantly smaller proportion of ÖGH (*n* = 2, 2.9%) and SGH (*n* = 1, 0.9%) members selected *Other* as their response to Q6 compared to DGH (*n* = 5, 17.2%) members (*p* = 0.012).

Moreover, a higher percentage of ÖGH members reported considering/treating both digital nerves than DGH members (44.3% vs. 21.1%, *p* = 0.004), while a greater proportion of DGH members (78.9% vs. 55.7%, *p* = 0.004) indicated considering/treating only the palmar digital nerves. No significant differences were observed for SGH members compared to other societies.

In terms of flexor tendon management, significantly more ÖGH members (20.0%) chose not to further shorten the tendons compared to DGH members (20.0% vs. 6.5%, *p* = 0.003), and a higher percentage of DGH members performed further flexor tendon shortening under axial traction than ÖGH and SGH members (89.8% vs. 71.4% vs. 72.4%, respectively, *p* = 0.003). Additionally, more ÖGH members opted for suturing the flexor tendon to the extensor tendon than members of the DGH and SGH (5.7% vs. 0.0% vs. 0.0%, *p* = 0.003).

Finally, more ÖGH members preferred tendon-to-bone fixation for extensor tendon management than their German-speaking counterparts (10.0% vs. 0.9%, *p* = 0.041) (Table 5).

### 3.5. Differences Based on Surgical Experience (≤20 Years vs. >20 Years)

Based on surgical experience, statistically significant differences were observed in the management of digital nerves (Q7), prevention of painful neuromas (Q8), and flexor tendon management (Q9) (Table 6).

Fewer hand surgeons with >20 years of experience reported further shortening the digital nerves (1.0%) compared to those with less experience (6.8%, *p* = 0.047). In contrast, more experienced surgeons were significantly more likely to reposition nerve endings away from the digital artery to prevent neuroma formation (12.8% vs. 0.0%, *p* = 0.034). Regarding flexor tendon management, experienced surgeons more often avoided additional tendon shortening (19.4% vs. 5.8%, *p* = 0.005), whereas less experienced surgeons more frequently employed the “pull and resect” technique (87.5% vs. 74.8%, *p* = 0.005).

## 4. Discussion

This survey study aimed to comprehensively assess surgical approaches for noninfected traumatic digital amputation injuries (excluding the thumb) at the level of the IP joints, focusing on techniques related to bone treatment, articular cartilage, digital nerves, prevention of painful neuromas, as well as flexor and extensor tendons. The questionnaire used in this study was adapted from Li et al. [8] to ensure comparability. Our study’s key finding highlights substantial differences in how surgical amputation is performed regarding disarticulation versus transosseous resection, reduction of the residual phalangeal head following disarticulation, digital nerve management, and prevention of painful neuromas.

### 4.1. Digital Nerve Management and Prevention of Painful Neuromas

We found more ÖGH members considering/treating both collateral nerves (44.3% vs. 21.1%, *p* = 0.004), while DGH members primarily consider/treat the palmar digital nerves (55.7% vs. 69.0%, *p* = 0.004). As most of our study population (69.9%) preferred the latter approach, it appears to be the preferred method. This can be explained by the fact that painful neuromas of the dorsal digital nerves are rarely reported, although a comparison with Li et al.’s study [8] is not possible as their questionnaire did not differentiate between dorsal and palmar digital nerves.

To prevent painful neuromas, it is generally advised to perform certain measures, including positioning the nerve ending away from areas that could potentially experience trauma, avoiding proximity to surgical incisions, keeping the nerve endings away from scar tissue, ensuring separation from the digital blood vessels, and performing a resection of the nerve that is as proximal and short as possible to facilitate the retraction of the nerve ending [8,32,33,34,35,36]. According to Li et al. [8], the preferred method for nerve management is resection via axial traction (92%), which aligns with our findings, indicating that most hand surgeons opt for the “pull and resect” technique for digital nerve treatment (54.8%). However, a notable proportion of our respondents (39.9%) reported performing specific techniques to prevent painful neuromas, with most of them placing the nerve endings away from high-pressure areas (44.2%) or the incision site (34.9%). These results are consistent with those of Li et al., who reported that 48% of American hand surgeons position the nerve endings away from expected repeated trauma areas, and an additional 49% place them away from the incision site (49%).

Neuroma excision, one of the oldest described surgical procedures to treat neuromas, has shown favorable outcomes in up to 68% of cases following traumatic digital amputation injuries [37]. Still, a higher revision rate has been observed compared to nerve repair (47% vs. 11%) [38]. Techniques like nerve stripping or thermal ablation have yielded higher incidences of painful neuromas (each 13%), while translocation of the nerve into the bone (0%) or centro-central union (CCU) of the proper two palmar digital nerves (0%) have shown better results [17,29,30]. Supporting these findings, Belcher and Pandya [28] observed no cases of symptomatic neuromas in their CCU group (0 out of 15) but two cases (2 out of 16) in their control group (nerve cut and cauterized). Similarly, a recent study by Maslow et al. [30] reported lower rates of postoperative neuroma formation following primary end-to-end nerve coaptation compared with traction neurectomy (12.8% vs. 22.7%) and found that persistent pain was significantly less common in patients undergoing coaptation (0% vs. 11.8%, *p* < 0.01). These findings further support the role of nerve coaptation techniques in reducing symptomatic neuroma formation after digital amputation. Consequently, CCU during surgical amputation appears to maximize the chances of preventing symptomatic neuromas.

Despite these favorable results, only a small percentage of respondents reported performing CCU (7.0%) or intraosseous transposition (7.0%) in our study. This may be explained by the acute trauma setting, where priorities include rapid wound management, tissue preservation, and minimization of operative time. In this context, CCU may be perceived as more technically demanding and time-consuming than simpler nerve management strategies. In addition, limited exposure during routine trauma practice and the lack of high-level evidence supporting clear superiority may further contribute to its infrequent use.

### 4.2. Miscellaneous

Functional aspects. Based on our findings, 97.6% of hand surgeons consider functional aspects in addition to the soft tissue condition and maximum length preservation. This aligns with the literature’s goal of preserving as much length as possible in a functional stump [39,40,41]. Consequently, when considering functional implications, it is crucial to consider the level of surgical amputation, as a non-functional stump could restrict the range of motion of adjacent fingers [42].

Disarticulation vs. transosseous resection. Regarding the treatment of bone in digital amputations at the level of the IP joints, the choice between disarticulation and resection through bone is a topic of discussion. Li et al. [8] found that 56% of American hand surgeons prefer disarticulation for amputation injuries, a tendency reflected in our findings, with 60.7% opting for disarticulation and 35.6% preferring the transosseous approach. Although IP disarticulation may have a potential advantage of lower symptomatic neuroma occurrence compared to transosseous bone resection at the finger phalanx level [29], the choice of transection depends on additional factors not addressed in this study.

Transosseous resection and bony edges. Li et al. [8] reported that 99% of American hand surgeons perform some form of bone edge smoothing as part of transosseous resection. Consistent with these findings, our study revealed a comparable percentage (98.8%) of hand surgeons who opted for smoothing bony edges after resection through bone. This approach is justified by its advantages, including the attainment of symmetrical stump ends, improved aesthetic outcome, and the prevention of pain and pressure sores [39,43].

Disarticulation and the phalangeal head. According to multiple studies [32,33,36,39], it appears to be generally agreed upon to reduce the size of the phalangeal head following IP joint disarticulation. Li et al. found that 82% of American hand surgeons perform phalangeal head reduction, whereas our survey results indicate a lower inclination towards phalangeal head reduction (63.3%) among respondents after disarticulation compared to American hand surgeons [8]. Both cosmetic and functional considerations likely drive the primary rationale for reducing the size of the residual phalangeal head. An unreduced phalangeal head can be extensive, aesthetically unappealing, and functionally inadequate, which can justify trimming the phalangeal head to achieve a smooth cone-shaped stump and ensure adequate soft tissue coverage [32,33,36].

Disarticulation and articular cartilage. The removal of the articular cartilage during disarticulation remains a topic of controversy, with some studies in favor of removal citing potential complications such as chondritis, necrosis, sequestration, and the development of a soft and sensitive stump [36,39,44]. Conversely, studies have shown that retaining the cartilage can result in fewer inflammatory reactions, improved stump flexibility, better length preservation, and more straightforward surgical procedures owing to reduced bleeding [45,46,47]. Furthermore, Conolly and Goulston proposed that the articular cartilage is anatomically designed to facilitate a pain-free force transmission [33]. Compared to the findings of Li et al. in 2013 [8], where 57% of hand surgeons remove the articular cartilage, our study indicates a stronger tendency towards cartilage removal (78.9%), unaffected by society affiliation or surgical experience. Recent clinical data indicate that whether the articular cartilage is removed or retained after joint disarticulation, there is no significant difference in infection rates, reoperation rates, or time to healing [48].The theoretical rationale for removing cartilage is to expose bleeding bone, which may promote granulation tissue formation and potentially reduce infection risk, but this has not been shown to confer a clear clinical advantage in practice [48,49]. Both approaches—removing the cartilage to expose bone or leaving the cartilage cap—are commonly practiced, and outcomes are comparable.

Flexor tendons. Whenever possible, preserving the insertions of the flexor tendons is generally recommended in the literature [32,36,39]. However, if preservation is feasible, the “pull and resect” technique is advised to avoid motion disorders, quadriga phenomena, and tendon imbalances [32,33,36,39,43,50]. In our study, 81.2% of participants reported using the “pull and resect” technique, reflecting this preference. This approach is particularly beneficial in amputation injuries at the level of the DIP joint with an uncertain length position, as shortening the flexor digitorum profundus (FDP) tendon under axial traction is more favorable than pulling it distally and fixing it in an extra-anatomical location. This preference is based on the understanding that quadriga phenomena have a more significant impact on function than the lumbricalis plus syndrome [51,52]. Furthermore, it is advised to avoid suturing the flexor and extensor tendons over the tip of or to the remnant bone due to potential impairments in tendon function [36,39,53,54]. According to our findings, only 1.9% of respondents reported performing this technique, which could be attributed to the potential cushioning effect at the bone tip.

Extensor tendons. Regarding the management of extensor tendons, the literature suggests that they should be shortened only to the level of the wound edge or slightly proximal to the level of the amputation [39,55]. Consistent with this recommendation, our results indicate a consensus in managing extensor tendons, with most hand surgeons (92.3%) not performing further shortening.

### 4.3. Limitations

First, the questionnaire utilized in this study employed single-choice and free-text response options to inquire about preferences, emphasizing that the provided choices should not be construed as absolute or definitive. It is important to note that the questionnaire did not allow for multiple responses, as the intention was to capture individual preferences. Accordingly, all survey responses should be interpreted as value-neutral preferences rather than “right” or “wrong” answers. Therefore, the single-choice responses included in the survey should be considered a limitation of the study, but from a legal perspective, they enable a differentiated assessment of tendencies. Second, by adopting the question from a previous study [8], this investigation did not consider critical confounding factors such as the exact level of amputation, patient factors, differentiation of FDS and FDP tendons, and more detailed injury characteristics that influence decision-making. However, this intentional decision was made to ensure the comparability of our findings with the data reported by Li et al. [8]. Furthermore, this study exhibits a very low response rate, possibly attributable to the lack of differentiation in the questionnaire and the timing of its distribution. The questionnaire focused more on an “ideal situation” with an “ideal patient”, inquiring about tendencies. Another reason for the relatively low response rate could be the decline in the number of digit amputation injuries in German-speaking countries due to the implementation of safety measures, including those in amateur equipment. Lastly, there is a geographical limitation as the questionnaire was explicitly addressed to members of the ÖGH, DGH, and SGH. However, this geographical limitation was intentional, which allowed comparing the results among experts in the three main German-speaking countries.

## 5. Conclusions

This study gathered valuable data regarding approaches to digital stump formation in digital amputation injuries (excluding the thumb) at the level of the IP joints. Based on our findings, we present an approach summarizing the techniques most commonly reported by hand surgeons in German-speaking countries surveyed in our study: Most consider functional aspects, along with soft tissue condition and preservation of maximum length. The preferred approach involves IP disarticulation, with articular cartilage removal and phalangeal head reduction. The palmar digital nerves are shortened under axial traction, and measures are taken to prevent symptomatic neuromas by relocating the nerve endings away from the anticipated point of highest pressure or incision site. Flexor tendons are shortened using the “pull and resect” technique, while extensor tendons do not receive specific treatment.

Overall, there is a consensus on most aspects, which mostly does not depend on nationality or surgical experience. Nevertheless, notable differences exist, specifically regarding digital nerve management and the prevention of neuromas. Therefore, future prospective studies should prioritise investigating these areas to uncover additional benefits.

## Figures and Tables

**Table 1:** The survey study’s questionnaire comprised a total of 10 questions (Q1–10) and included intelligent routing to facilitate subquestions related to specific subtopics. All experts were asked Q1–2, Q6–7, and Q9–10. Q3–5 and Q8 appeared as additional questions depending on the given response (intelligent routing).

Question (Q)		Answer Options
Q1	“Do you perform surgical digit amputation solely considering the soft tissue situation and aiming for maximum length preservation, or do you also consider functional aspects?”	Considering the soft tissue situation and aiming for maximum length preservation with consideration of functional aspects
		Considering the soft tissue situation and aiming for maximum length preservation without consideration of functional aspects
Q2 ^a^	“Which of the specified techniques for surgical digit amputation do you perform for non-infected traumatic amputation injuries at the level of the interphalangeal joints (IP)?”	Disarticulation
		Resection through bone (transosseous resection)
		Other
Q3 *	“Do you perform an additional smoothing of the bone edges during resection through bone?”	No
		Yes
Q4 *	“Do you remove the articular cartilage during disarticulation?”	No
		Yes
Q5 *	“Do you reduce the size of the residual phalangeal head during disarticulation?”	No
		Yes
Q6	“Do you consider/treat only the palmar digital nerves or also the dorsal digital nerves during surgical digit amputation?”	Only the palmar digital nerves
		Only the dorsal digital nerves
		Both
Q7 ^b^	“How do you treat the digital nerves of the affected fingers?”	No further shortening
		Further shortening of the nerves under axial traction (“pull and resect”)
		Specific techniques for the prevention of painful neuromas
		Other
Q8 *	“Which of the following techniques do you use to prevent painful neuromas during surgical digit amputation?”	Placing nerve endings away from expected highest point of pressure (equivalent to the fingertip)
		Placing nerve endings away from incision site
		Placing nerve endings away from digital arterial blood vessel
		Intraosseous nerve transposition
		Nerve coaptation
		Other (nerve resection)
Q9	“How do you manage the flexor tendons?”	No further shortening
		Further shortening under axial traction (“pull and resect”)
		Tendon-to-bone fixation
		Suturing to the extensor tendon stump
		Other
		No further shortening
Q10	“How do you manage the extensor tendons?”	Tendon-to-bone fixation
		Suturing to the flexor tendon stump
		Other

^a^ If *disarticulation* was chosen as the answer for Q2, Q4 and Q5 needed to be answered additionally. However, if Q2 was responded to with *resection through bone* or *other*, Q3 was to be answered additionally. ^b^ For Q7, the option *Specific techniques for preventing painful neuromas* required answering Q8 as well. * Questions asked via intelligent routing.

**Table 2:** Characteristics of the study population (*n* = 213).

Parameter		*n* (%)
Age group [in years]	<30	1 (0.5)
	30–35	41 (19.3)
	40–49	72 (33.8)
	50–59	61 (28.6)
	≥60	38 (17.8)
Gender	Male	161 (75.6)
	Female	52 (24.4)
Affiliated society	ÖGH	71 (33.3)
	DGH	112 (52.6)
	SGH	30 (14.1)
Surgical experience [in years]	0–5	5 (2.4)
	6–10	31 (14.6)
	11–20	72 (33.8)
	>20	105 (49.3)

ÖGH, Österreichische Gesellschaft für Handchirurgie (Austrian Society for Surgery of the Hand, ÖGH); DGH, Deutsche Gesellschaft für Handchirurgie (German Society for Hand Surgery, DGH); SGH, Schweizerische Gesellschaft für Handchirurgie (Swiss Society for Hand Surgery, SGH).

**Table 3:** Differences in demographic characteristics based on affiliated society.

	ÖGH		DGH		SGH		*p* Value
	*n*	% *	*n*	% *	*n*	% *	
Age group [in years]							0.348 ^a^
<30	1	1.4%	0	0.0%	0	0.0%	
30–39	15	21.4%	19	17.4%	5	17.2%	
40–49	18	25.7%	40	36.7%	12	41.4%	
50–59	18	25.7%	34	31.2%	9	31.0%	
≥60	18	25.7%	16	14.7%	3	10.3%	
Gender							0.808 ^a^
Male	53	75.7%	85	78.0%	21	72.4%	
Female	17	24.3%	24	22.0%	8	27.6%	
Surgical experience [in years]							**0.014 ^b^**
0–5	4	5.7%	1	0.9%	0	0.0%	
6–10	14	20.0%	12	11.0%	4	13.8%	
11–20	13	18.6%	44	40.4%	13	44.8%	
>20	39	55.7%	52	47.7%	12	41.4%	

* Percentage of respective society. ^a^ Pearson chi-square test. ^b^ Fisher’s exact test. **Bold** = *p* value < 0.05 regarding the respective question. ÖGH, Österreichische Gesellschaft für Handchirurgie (Austrian Society for Surgery of the Hand, ÖGH); DGH, Deutsche Gesellschaft für Handchirurgie (German Society for Hand Surgery, DGH); SGH, Schweizerische Gesellschaft für Handchirurgie (Swiss Society for Hand Surgery, SGH).

**Table 4:** Number and percent response per question (Q1–10).

Question	Allowed [*n*]	Responded [*n*]	Response Rate [%]	Skipped [*n*]	Not Surveyed [*n*] *
Q1	213	211	99.1%	2	0
Q2	213	211	99.1%	2	0
Q3	83	83	100%	0	130
Q4	128	128	100%	2	83
Q5	128	128	100%	2	83
Q6	213	209	98.1%	4	0
Q7	213	208	97.7%	5	0
Q8	86	86	100%	0	127
Q9	213	207	97.2%	6	0
Q10	213	207	97.2%	6	0

* Based on the answers in questions 2 and 7 (Q2 and Q7), additional questions (Q3–5 and Q8) had to be answered following intelligent routing. A detailed description of the question’s sequences is provided in the Section 2 (Table 1).

**Table 5:** Responses stratified by affiliated society.

		ÖGH		DGH		SGH		*p* Value
Q1								0.201 ^b^
	Considering the soft tissue situation and aiming for maximum length preservation with consideration of functional aspects	70	100%	105	96.3%	29	100%	
	Considering the soft tissue situation and aiming for maximum length preservation without consideration of functional aspects	0	0.0%	4	3.7%	0	0.0%	
Q2								**0.012 ^b^**
	Disarticulation	41	58.6%	71	65.1%	15	51.7%	
	Resection through bone (transosseous resection)	27	38.6%	37	33.9%	9	31.0%	
	Other	2	2.9%	1	0.9%	5	17.2%	
Q3								1.000 ^b^
	No	0	0.0%	1	2.6%	0	0.0%	
	Yes	29	100%	37	97.4%	14	100%	
Q4								0.148 ^a^
	No	5	11.9%	17	23.9%	5	33.3%	
	Yes	37	88.1%	54	76.1%	10	66.7%	
Q5								0.077 ^a^
	No	12	28.6%	32	45.1%	3	20.0%	
	Yes	30	71.4%	39	54.9%	12	80.0%	
Q6								**0.004 ^a^**
	Only the palmar digital nerves	39	55.7%	86	78.9%	20	69.0%	
	Only the dorsal digital nerves	0	0.0%	0	0.0%	0	0.0%	
	Both	31	44.3%	23	21.1%	9	31.0%	
Q7								0.066 ^b^
	No further shortening	2	2.9%	6	5.5%	0	0.0%	
	Further shortening of the nerves under axial traction (“pull and resect”)	47	67.1%	55	50.5%	12	41.4%	
	Specific techniques for the prevention of painful neuromas	20	28.6%	47	43.1%	16	55.2%	
	Other	1	1.4%	1	0.9%	1	3.5%	
Q8								0.050 ^b^
	Placing nerve endings away from expected highest point of pressure (equivalent to the fingertip)	14	66.7%	14	29.2%	10	58.8%	
	Placing nerve endings away from incision site	3	14.3%	20	41.7%	7	41.2%	
	Placing nerve endings away from digital arterial blood vessel	0	0.0%	5	10.4%	0	0.0%	
	Intraosseous nerve transposition	2	9.5%	4	8.3%	0	0.0%	
	Nerve coaptation	2	9.5%	4	8.3%	0	0.0%	
	Other (nerve resection)	0	0.0%	1	2.1%	0	0.0%	
Q9								**0.003 ^b^**
	No further shortening	14	20.0%	7	6.5%	5	17.2%	
	Further shortening under axial traction (“pull and resect”)	50	71.4%	97	89.8%	21	72.4%	
	Tendon-to-bone fixation	2	2.9%	3	2.8%	2	6.9%	
	Suturing to the extensor tendon stump	4	5.7%	0	0.0%	0	0.0%	
	Other	0	0.0%	1	0.9%	1	3.5%	
Q10								**0.041 ^b^**
	No further shortening	60	85.7%	103	95.4%	28	96.6%	
	Tendon-to-bone fixation	7	10.0%	1	0.9%	0	0.0%	
	Suturing to the flexor tendon stump	2	2.9%	1	0.9%	0	0.0%	
	Other	1	1.4%	3	2.8%	1	3.5%	

^a^ Pearson chi-square test. ^b^ Fisher’s exact test. Q1–10, Question 1–10 of the questionnaire; ÖGH, Österreichische Gesellschaft für Handchirurgie (Austrian Society for Surgery of the Hand, ÖGH); DGH, Deutsche Gesellschaft für Handchirurgie (German Society for Hand Surgery, DGH); SGH, Schweizerische Gesellschaft für Handchirurgie (Swiss Society for Hand Surgery, SGH). **Bold** = *p* value < 0.05 regarding the respective question.

**Table 6:** Distribution of the number of respondents and response rates.

		≤20 Years of Surgical Experience		>20 Years of Surgical Experience		*p* Value
		*n*	% *	*n*	% **	
Q1						1.000 ^a^
	Considering the soft tissue situation and aiming for maximum length preservation with consideration of functional aspects	103	98.1%	101	98.1%	
	Considering the soft tissue situation and aiming for maximum length preservation without consideration of functional aspects	2	1.9%	2	1.9%	
Q2						0.341 ^a^
	Disarticulation	68	64.8%	59	57.3%	
	Resection through bone (transosseous resection)	32	30.5%	41	39.8%	
	Other	5	4.8%	3	2.9%	
Q3						0.457 ^a^
	No	1	2.7%	0	0.0%	
	Yes	36	97.3%	44	100%	
Q4						0.078 ^a^
	No	10	14.5%	17	28.8%	
	Yes	59	85.5%	42	71.2%	
Q5						0.669 ^b^
	No	27	39.1%	20	33.9%	
	Yes	42	60.9%	39	66.1%	
Q6						0.319 ^b^
	Only the palmar digital nerves	39	55.7%	86	78.9%	
	Both	31	44.3%	23	21.1%	
Q7						**0.047 ^a^**
	No further shortening	1	1.0%	7	6.8%	
	Further shortening of the nerves under axial traction (“pull and resect”)	57	54.3%	57	55.3%	
	Specific techniques for the prevention of painful neuromas	44	41.9%	39	37.9%	
	Other	3	2.9%	0	0.0%	
Q8						**0.034 ^a^**
	Placing nerve endings away from expected highest point of pressure (equivalent to the fingertip)	25	53.2%	13	33.3%	
	Placing nerve endings away from incision site	17	36.2%	13	33.3%	
	Placing nerve endings away from digital arterial blood vessel	0	0.0%	5	12.8%	
	Intraosseous nerve transposition	2	4.3%	4	10.3%	
	Nerve coaptation	2	4.3%	4	10.3%	
	Other (nerve resection)	1	2.1%	0	0.0%	
Q9						**0.005 ^a^**
	No further shortening	6	5.8%	20	19.4%	
	Further shortening under axial traction (“pull and resect”)	91	87.5%	77	74.8%	
	Tendon-to-bone fixation	2	1.9%	5	4.9%	
	Suturing to the extensor tendon stump	3	2.9%	1	1.0%	
	Other	2	1.9%	0	0.0%	
Q10						0.761 ^a^
	No further shortening	97	93.3%	94	91.3%	
	Tendon-to-bone fixation	3	2.9%	5	4.9%	
	Suturing to the flexor tendon stump	1	1.0%	2	1.9%	
	Other	3	2.9%	2	1.9%	

* Percentage of respondents with surgical experience ≤ 20 years. ** Percentage of respondents with surgical experience > 20 years. ^a^ Pearson chi-square test. ^b^ Fisher’s exact test. Q1–14, Question 1–14 of the questionnaire; ÖGH, Österreichische Gesellschaft für Handchirurgie (Austrian Society for Surgery of the Hand, ÖGH); DGH, Deutsche Gesellschaft für Handchirurgie (German Society for Hand Surgery, DGH); SGH, Schweizerische Gesellschaft für Handchirurgie (Swiss Society for Hand Surgery, SGH). **Bold** = *p* value < 0.05 regarding the respective question.

## Data Availability

The raw data supporting the conclusions of this article will be made available by the authors on request.

## Abbreviations

The following abbreviations are used in this manuscript:

DGH	Deutsche Gesellschaft für Handchirurgie (German Society for Hand Surgery, DGH);
DIP	Distal interphalangeal joint
FDP	Flexor digitorum profundus
FDS	Flexor digitorum superficialis
IP	Interphalangeal joint
ÖGH	Österreichische Gesellschaft für Handchirurgie (Austrian Society for Surgery of the Hand)
PIP	Proximal interphalangeal joint
SGH	Schweizerische Gesellschaft für Handchirurgie (Swiss Society for Hand Surgery)
